# Influence of Hematocrit and Total-Spot Volume on Performance Characteristics of Dried Blood Spots for Newborn Screening

**DOI:** 10.3390/ijns1020069

**Published:** 2015-08-21

**Authors:** Elizabeth M. Hall, Sharon R. Flores, Víctor R. De Jesús

**Affiliations:** Newborn Screening and Molecular Biology Branch, Division of Laboratory Sciences, National Center for Environmental Health, US Centers for Disease Control and Prevention, 4770 Buford Highway, NE, Mail Stop F-19, Atlanta, GA 30341, USA

**Keywords:** dried blood spots, newborn screening, hematocrit, filter paper, specimen collection device

## Abstract

Dried blood spots (DBS) have been used in newborn screening (NBS) tests for over 50 years. The Newborn Screening Quality Assurance Program (NSQAP) at the Centers for Disease Control and Prevention (CDC) conducted studies to assess the individual impacts of hematocrit and total-spot volume on characteristics of DBS samples. Per-punch serum volumes decreased 27%, RBC volumes more than doubled, absorption times increased over 300%, and spot diameters decreased marginally between the hematocrits of 40% to 65%. Per-punch serum and RBC volumes decreased logarithmically with lowering total-spot volumes. Patient hematocrit is an uncontrollable variable and inevitably affects the resulting punch from a DBS sample. It may be possible, though, to identify samples that fall outside of an acceptable range by noting certain physical characteristics of the DBS.

## 1. Introduction

Newborn screening (NBS) began in the early 1960s when Dr. Robert Guthrie developed an assay that used bacterial growth patterns to test small disks of blood-saturated blotter paper for elevated levels of phenylalanine [1]. Over 50 years later, dried blood spot (DBS) samples are still collected from babies’ heels in much the same way, but the filter paper used is more stringently regulated and NBS testing methods have made dramatic advances. Today’s testing methods are almost exclusively quantitative—requiring a consistent sample volume in order to deliver comparable results—and NBS laboratories rely on the assumption that identically sized DBS punches contain relatively uniform volumes of blood. To minimize any lot-to-lot variations, filter paper approved by the US Food and Drug Administration (FDA) for use as NBS DBS collection devices is made according to strict specifications and must perform within the parameters detailed in the Clinical and Laboratory Standards Institute (CLSI) document NBS01-A6, Blood Collection on Filter Paper for Newborn Screening Programs; Approved Standard—Sixth Edition [2]. The Newborn Screening Quality Assurance Program (NSQAP) at the Centers for Disease Control and Prevention (CDC) performs an evaluation of each new lot to assess its blood absorption characteristics using 55% hematocrit, washed-cell blood and a 100 μL per-DBS volume [3]. Under these controlled conditions, the thirty FDA-approved lots that NSQAP tested during the 10-year period from 2004 to 2014 showed a mean serum absorption volume of 1.46 μL per 3.2 mm punch with a standard deviation of 0.05 μL. However, despite the consistency of the collection matrix, varying hematocrits and total spot volumes can still result in identically sized punches that contain dissimilar blood and serum volumes.

The hematocrit range (5th to 95th percentile) for full-term newborns is 42% to 65%; premature infants have slightly lower values [4]. In addition to the effects from hematocrit, the total volume of blood used to create a DBS can also affect resulting punch volume [5]. The CLSI Approved Standard NBS01-A6 specifies that the printed circles on NBS DBS collection cards have an inner diameter of 12–13 mm, and states that 75 μL of blood will just fill the area while 100 μL will fill just beyond the print. These printed circles serve as a guide to the specimen collector and help to ensure that neither too little nor too much blood is applied.

Efforts to find a DBS analyte that can serve as a correction factor for punch volume from DBS of patients with different hematocrits have been documented [6], as have attempts to determine the best way to prepare DBS quality control materials with appropriate hematocrits [7]. However, few studies have specifically addressed the issues faced by NBS laboratories. Most of these studies have been aimed at serving the pharmaceutical development community where DBS have become an increasingly popular way of collecting samples during therapeutic drug monitoring (TDM) [8–12]. Although it would not be feasible to evaluate each lot for all possible patient hematocrits and DBS volumes, we present the results here from studies conducted to assess their individual impacts.

We aimed to answer the following questions, with a focus on the range of specimen variability encountered by newborn screening laboratories:

- How much does blood hematocrit impact the resulting whole blood, serum, and red blood cell (RBC) volume of the resulting punches?
- How much does total spot volume impact punch volume?
- Are there any visual cues that a screening laboratory might be able to use to assess whether a DBS might fall outside of an acceptable range?

## 2. Experimental Section

### 2.1. Materials

Grade 226 and Grade 903 filter papers were obtained from PerkinElmer Health Sciences (Greenville, SC, USA) and GE Healthcare Bio-Sciences Corporation (Westborough, MA, USA), respectively. Human serum and human packed red blood cells (RBCs) collected in CPDA -1 anticoagulant were purchased from Tennessee Blood Services (Memphis, TN, USA). Blood bank saline (0.9% sodium chloride) was from Fisher Scientific (Waltham, MA, USA). High specific activity iodine-125-labeled L-thyroxine (^125^I-labeled T_4_) was obtained from PerkinElmer Radiochemicals (Billerica, MA, USA). For serum filtration, we used disposable Nalgene filtration units (cellulose nitrile membrane, 0.2 μm pore size) from Fisher Scientific.

### 2.2. Methods

**Figure 1:**
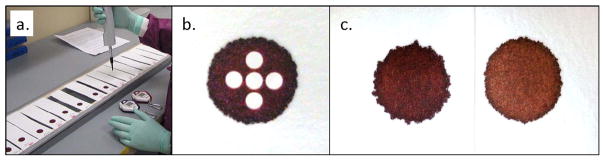
Paper suspension system, punch configuration, and hematocrit-dependent appearance differences. (**a**) Sample strips of filter paper are suspended horizontally across metal racks using double-stick tape; (**b**) Five 3.2 mm diameter punches were sampled from each DBS in a center, north, east, south, west configuration; (**c**) On the left, a 40% hematocrit, 100 μL spot. On the right, a 65% hematocrit, 100 μL spot.

We used the procedure outlined in CLSI Standard NBS01-A6, Appendix C [2]. All filter paper samples were cut into strips approximately 15 cm long and 4 cm wide. All strips were blind-coded and randomized before being suspended horizontally across metal frames, using double-stick tape to secure each end (Figure 1a). The strips were then allowed to acclimate to ambient conditions for at least 24 h before blood application.

All blood used for these experiments was saline-washed RBCs combined with filtered serum. The RBCs were washed three times by combining with an approximately equal volume of saline, centrifuging for separation, and then removing saline and buffy coat by suction. The first two spins were at 3200 RPM (2910 *g*) for 10 min and the last spin was at 4000 RPM (4550 *g*) for 15 min. The hematocrit after the final saline removal was >90%. Calculated volumes of filtered serum were added to measured volumes of washed RBCs to achieve desired hematocrits. The blood used for the total spot volume study was at a hematocrit of 55%. The six pools used in our hematocrit comparison studies were at 40%, 45%, 50%, 55%, 60%, and 65% ± 1%. The hematocrit of each blood pool was analytically verified before use.

Blood aliquots were dispensed onto filter paper using electronic repeater pipettes with positive-displacement, syringe-style tips. Absorption times were measured using a handheld stopwatch that was started when the blood touched the paper and stopped when any shine from liquid blood was no longer visible on the surface. Diameters of the dried spots were measured with an electronic micro-caliper across the width and length of each spot.

Volume-per-punch data were derived by using blood enriched with ^125^I-labeled T_4_. Blood pools were enriched to an activity level of 1.6 μCi/mL and dispensed onto the suspended paper strips in the volumes indicated. For each pool, one 100 μL aliquot of blood was also added to each of five 10 mL portions of saline to create 1:100 dilutions. After an overnight drying period, 3.2 mm punches were sampled from each isotopically enriched DBS using an automated puncher. For the hematocrit study where all spots were 100 μL, five punches were taken from each DBS in a center, north, east, south, west configuration (Figure 1b). For the spot volume study, one 3.2 mm punch was sampled from the center of each spot. All DBS punches were then assayed on a PerkinElmer WIZARD2 2470 gamma counter. At the same time, 400 μL samples—containing 4.0 μL of whole blood—from each liquid dilution were also assayed and used as calibrators to determine the volume of dried liquid blood contained in the DBS punches.

For each variable we investigated, more than one lot of filter paper was tested. Because lot-to-lot differences were minimal, we present the composite data from each experiment.

## 3. Results and Discussion

### 3.1. Effects of Hematocrit on Punch Volumes, Absorption Times, and Spot Diameters

**Figure 2:**
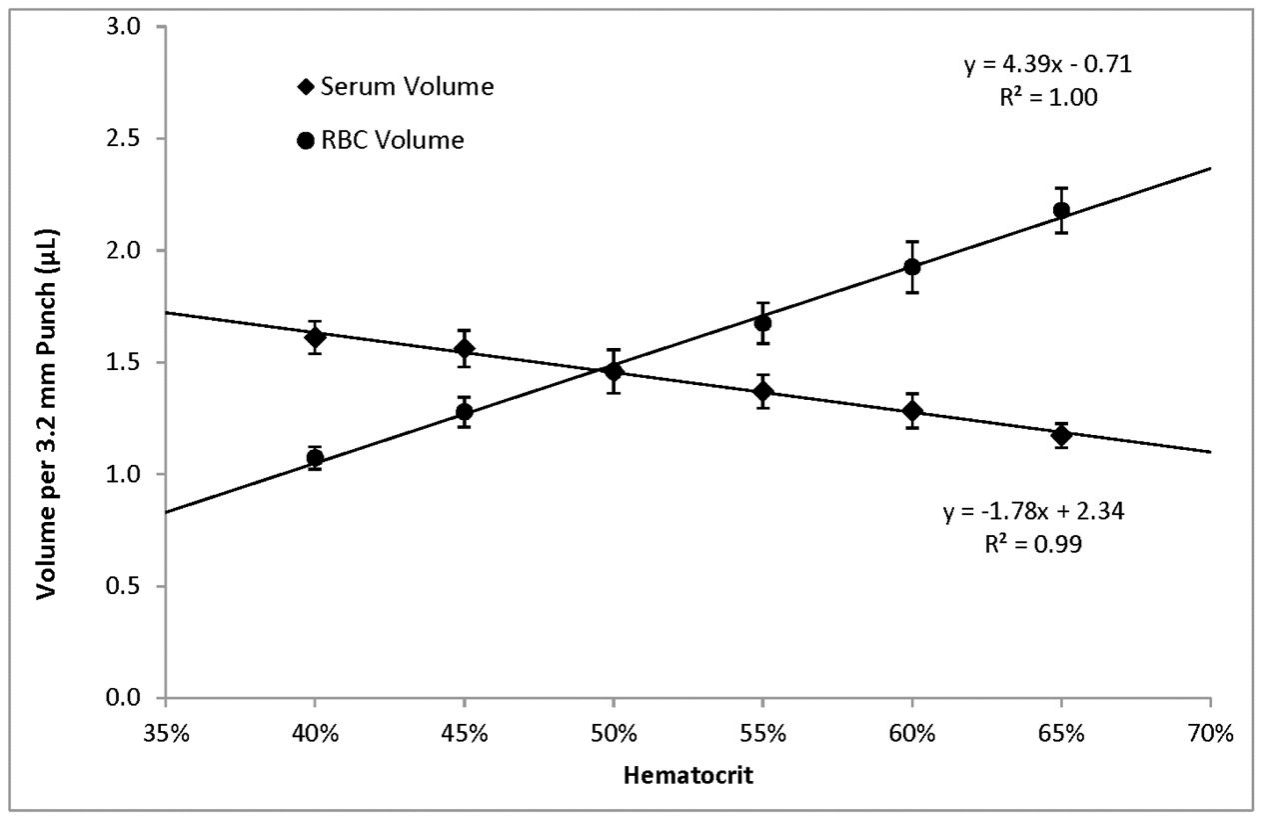
Average serum and RBC volumes contained in 3.2 mm punches sampled from 100 μL DBS of different hematocrits. Error bars represent ±1 SD, *n* = 50 (5 punches from each of 10 DBS).

**Figure 3:**
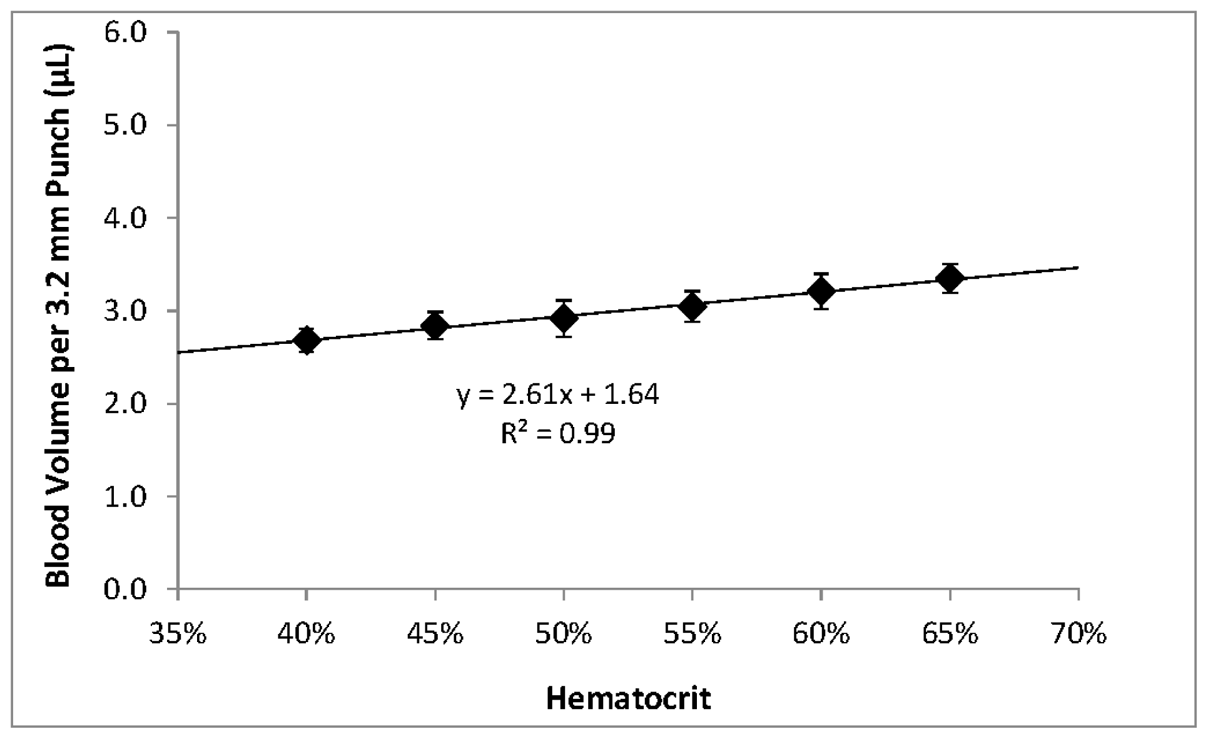
Average whole blood volumes contained in 3.2 mm punches sampled from 100 μL DBS of different hematocrits. Error bars represent ±1 SD, *n* = 50 (5 punches from each of 10 DBS).

Within the range of hematocrits that we investigated, higher hematocrits resulted in higher per-punch volumes of whole blood and RBCs but lower per-punch volumes of serum. RBC volume appeared to be the larger contributor to hematocrit-dependent changes in per-punch whole blood volume; serum volumes showed less variation over the range of hematocrits. When hematocrit increased from 40% to 65%, per-punch serum volume decreased by 27% while per-punch RBC volume more than doubled (Figure 2). Per-punch whole blood volume increased 25% over the range (Figure 3).

As hematocrit decreases, the serum component of the blood increases. Both blood components—serum and RBCs—increased in per-punch volume as their relative percentage of whole blood increased (Figure 2). This may help explain why per-punch whole blood volumes changed less than either component alone (Figure 3).

**Figure 4:**
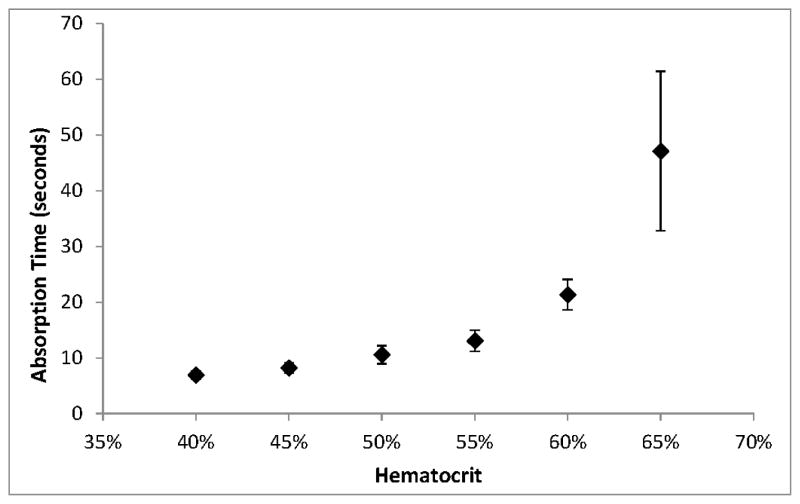
Average absorption times for 100 μL aliquots of different hematocrits. Error bars represent ±1 SD, *n* = 15.

As we have observed before [13], absorption time increased with hematocrit, with the most significant changes at hematocrits above 55%. The CLSI Approved Standard NBS01-A6 states that the acceptable range for absorption of 100 μL of 55% hematocrit blood is 5 to 30 s. At 65% hematocrit, 100 μL aliquots exceeded the upper end of this range (Figure 4).

**Figure 5:**
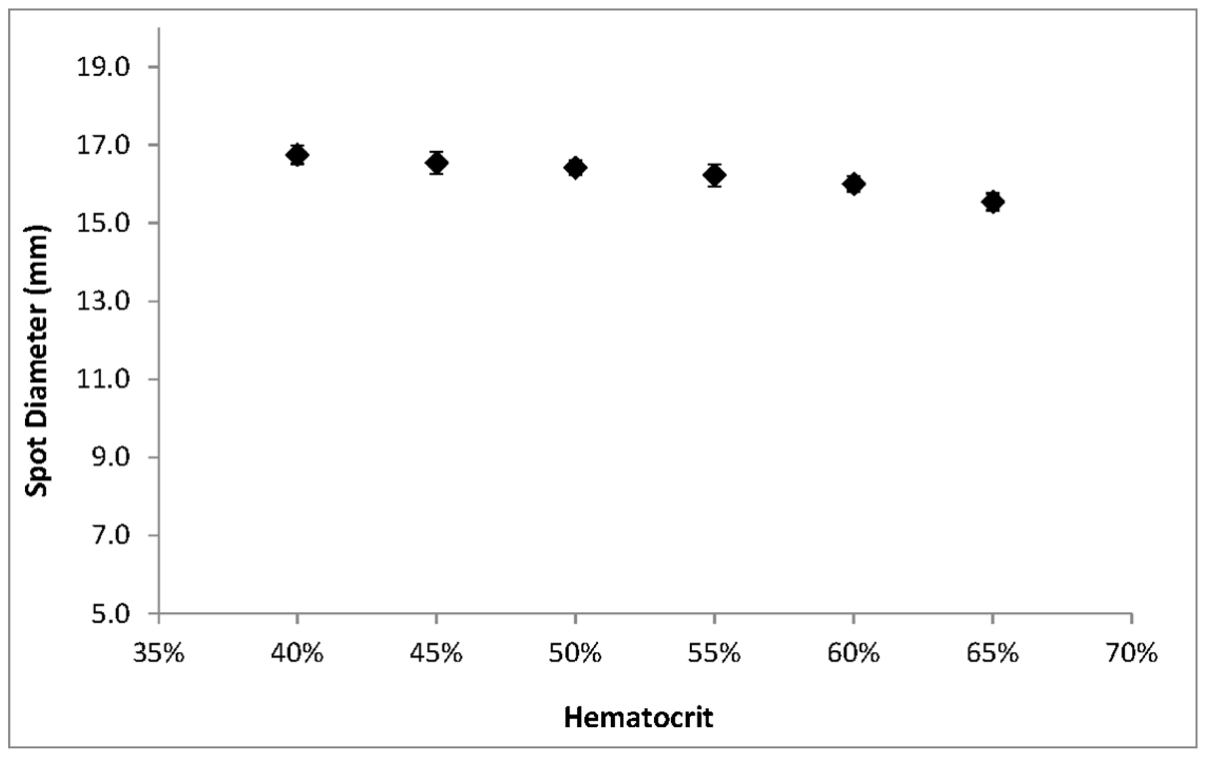
Average diameter for 100 μL DBS of different hematocrits. Error bars represent ±1 SD, *n* = 15.

Among the hematocrit-dependent features that we investigated, spot diameters showed the smallest relative variations across the range. Spots were larger at low hematocrits but decreased an average of only 1.2 mm—less than 8%—over the full range of hematocrits (Figure 5). The appearance of spots also changes with hematocrit; a lower concentration of RBCs results in a more uniform, smooth edge while more viscous, higher-hematocrit blood causes the spots to have a more uneven, jagged outline (Figure 1c).

### 3.2. Effects of Spot Volumes on Punch Volumes and Spot Diameters

**Figure 6:**
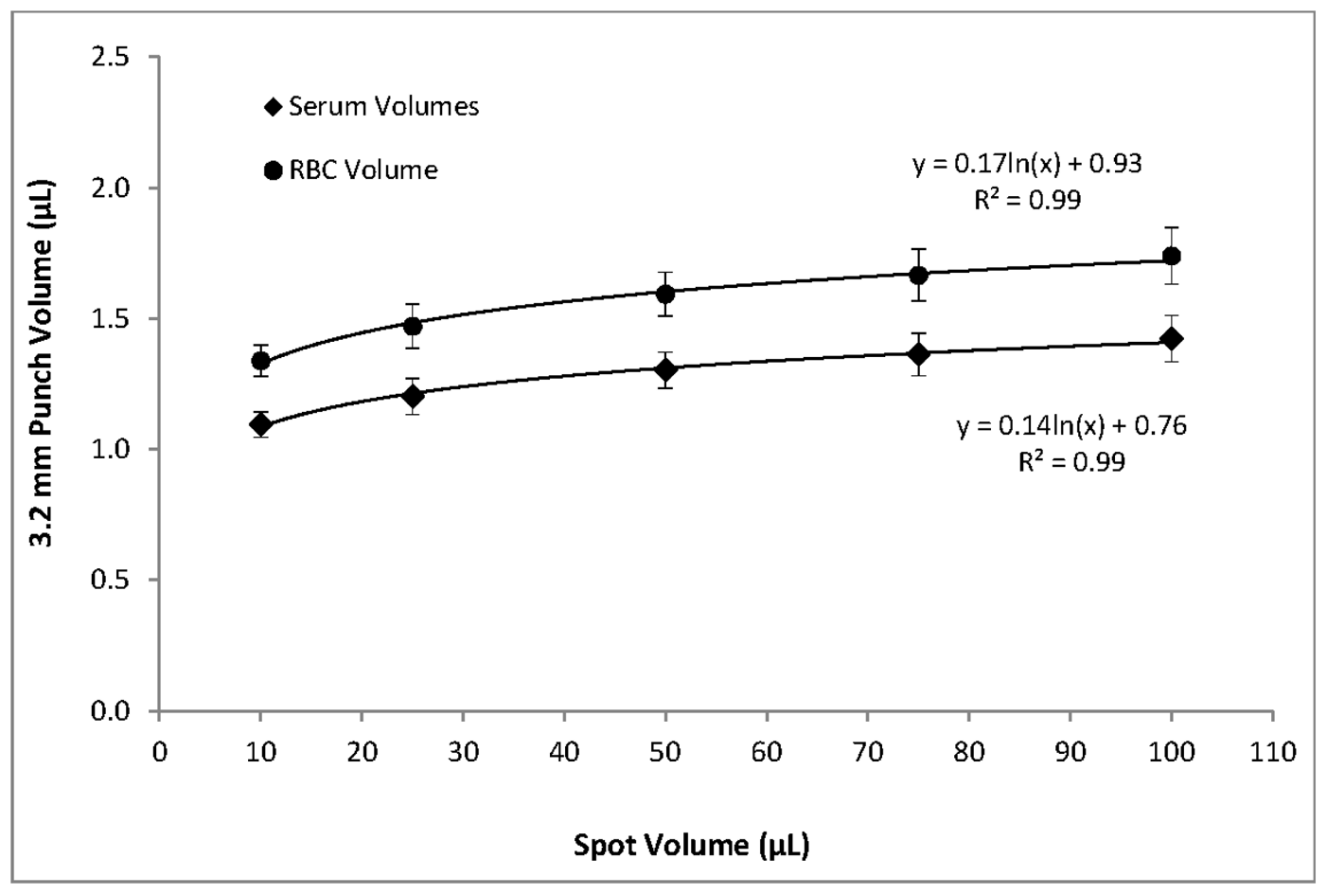
Average per-punch serum and RBC volumes for 55% hematocrit DBS of different total volumes. Due to spot size limitations, only center punches were used for this study. Error bars represent ±1SD, *n* = 75.

**Figure 7:**
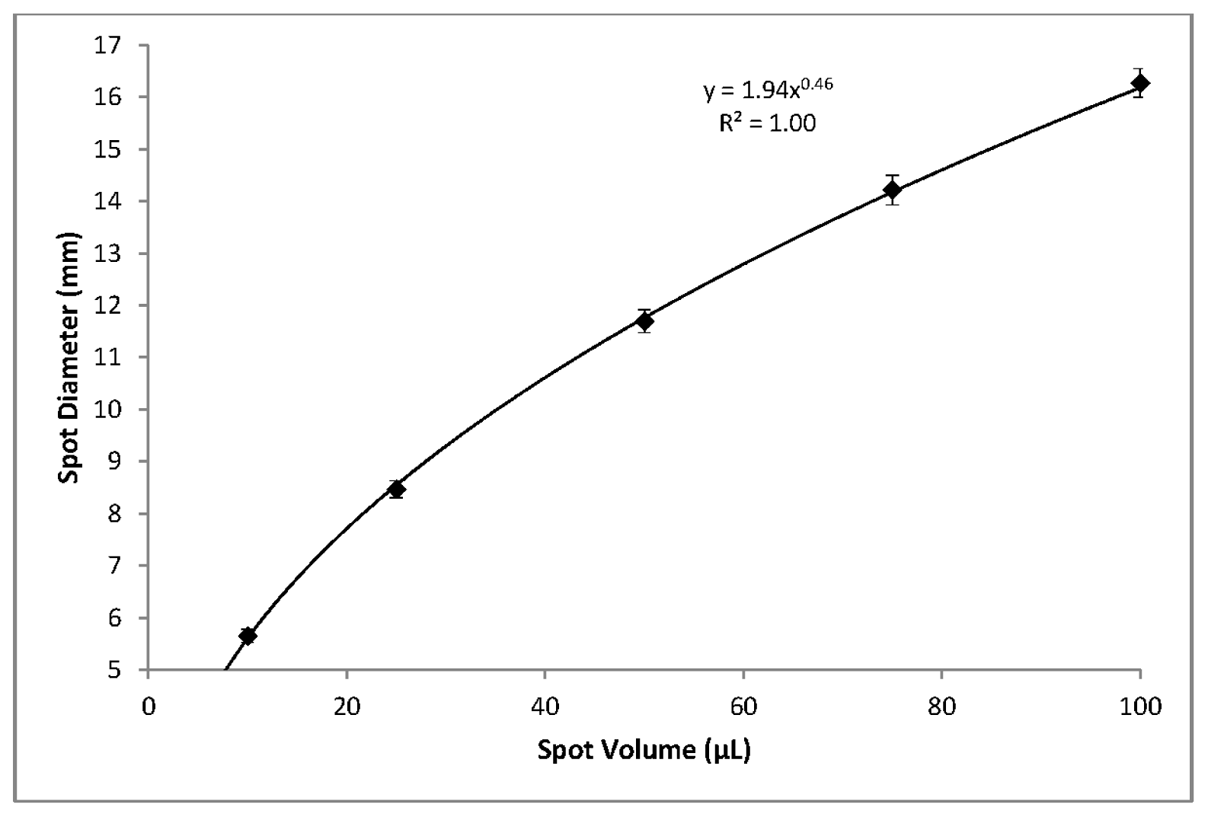
Average diameter for DBS of different total volumes. Error bars represent ±1 SD, *n* = 75.

Using blood with a 55% hematocrit, the effects of spot volume alone on per-punch volumes of serum, RBCs, and whole blood followed a logarithmic pattern. A reduction in spot volume from 100 to 75 μL resulted in 3.2 mm punches with 4% lower volumes of serum, RBCs, and whole blood. Reducing spot volume to 50 μL resulted in an additional 4% loss of per-punch volume. Further lowering spot volume to 25 μL lowered punch volumes 8% more and 10 μL spots yielded punches with 23% less volume than those from 100 μL spots (Figure 6). It should be noted that a reduction in spot volume from 100 μL to not less than 25 μL resulted in a 16% loss of per-punch sample volume. Similarly, spot volume effects on spot diameters were logarithmic (Figure 7). As expected, the relationship between spot volume and spot diameter was consistent with the mathematical relationship between circle area and circle diameter. In our experiments, spot diameter could be consistently predicted by the following equation:

$$\mathrm{Diameter}\;in\;mm=2\times \sqrt{\frac{\frac{\mathrm{spot}\;\mathrm{volume}\;in\;\mu L}{0.4918}+5.314}{\pi }}$$

## 4. Conclusions

The use of DBS in newborn screening has opened the doors to a field of study that deserves the attention it is receiving in current literature [14]. In the United States, only filter papers from sources cleared by the FDA are acceptable for blood collection for newborn screening tests. An ongoing assessment and evaluation of new production lots as they are manufactured is critical to proper and effective use of this matrix, as well as the monitoring of problems identified with individual lots in use by NBS programs [3]. Among the variables we investigated, screening laboratories’ paramount concern is the resulting sample volume. Without consistent specimens, patient-to-patient comparable results are unattainable.

Our results indicate hematocrit has less of an impact on per-punch serum volumes than RBC volumes and that per-punch whole blood volumes changed less than either component alone (Figures 2 and 3). Because of this, RBC-associated analytes—such as galactose-1-phosphate uridyltransferase—may show greater variability than serum-based analytes. While the hematocrit of a newborn’s blood cannot be controlled, our studies suggest that there may be certain indicators that both specimen collectors and the receiving screening laboratories can use to their advantage.

DBS diameter is greatly influenced by total spot volume (Figure 7) but is not highly influenced by hematocrit (Figure 5). Per-punch blood volumes and spot diameters both show a logarithmic relationship to total spot volumes and, interestingly, a linear relationship to one another. CLSI Standard NBS01-A6 states that printed circles on filter paper collection cards should have an internal diameter of 12 to 13 mm and that 75 μL will just fill the circle while 100 μL will fill slightly beyond the print [2]. Printed circles of the prescribed diameter serve as literal guidelines to help specimen collectors obtain samples with adequate volume. The printed lines also allow screening laboratories to discern—through a brief visual inspection—that a specimen’s total-spot volume is substantially below the target of 75 to 100 μL and avoid spots whose per-punch volumes may fall outside an acceptable range.

In addition to any disparity between a spot’s diameter and the printed circle, there are other visual cues that may indicate an unusual specimen. Lower-hematocrit blood produces spots with noticeably smoother edges while the borders of higher-hematocrit spots appear more irregular (Figure 1c). Laboratory personnel who become accustomed to normal spot appearance could conceivably discern specimens with hematocrits at either extreme, based on visual inspection. Coupled with a spot-diameter to printed-line comparison, a visual inspection of spot-edge appearance could serve to identify specimens with compound problems (*i.e.*, both total-spot volume and hematocrit) that, together, may significantly impact the resulting punched samples.

Our investigations are limited by the fact that we must use washed RBCs with serum, a matrix devoid of the clotting factors present in heel-stick blood from newborns. It is not known whether this difference significantly impacted our results. Nevertheless, this study presents a standardized approach to evaluate the filter paper matrix’s absorption characteristics. Hematocrit effect evaluations for TDM and other bioanalysis applications may benefit from over 30 years of experience with the use of DBS samples for population-based screening.

Filter paper used in specimen collection devices (cards) by newborn screening programs has exhibited stable performance characteristics for over 30 years. Nevertheless, issues with hematocrit and sample collection remain a challenge to the widespread use of filter paper blood collection devices. As filter paper matrices become more common in bioanalysis, we expect that analyte-specific performance characteristics will be determined in a standardized manner, enabling harmonization of practices throughout the field.
